# Multiple exaggerated weapon morphs: a novel form of male polymorphism in harvestmen

**DOI:** 10.1038/srep16368

**Published:** 2015-11-06

**Authors:** Christina J. Painting, Anna F. Probert, Daniel J. Townsend, Gregory I. Holwell

**Affiliations:** 1 School of Biological Sciences, University of Auckland, Private Bag 92019, Auckland Mail Centre, Auckland 1142, New Zealand

## Abstract

Alternative reproductive tactics in animals are commonly associated with distinct male phenotypes resulting in polymorphism of sexually selected weapons such as horns and spines. Typically, morphs are divided between small (unarmed) and large (armed) males according to one or more developmental thresholds in association with body size. Here, we describe remarkable weapon trimorphism within a single species, where two exaggerated weapon morphs and a third morph with reduced weaponry are present. Male *Pantopsalis cheliferoides* harvestmen display exaggerated chelicerae (jaws) which are highly variable in length among individuals. Across the same body size spectrum, however, some males belong to a distinct second exaggerated morph which possesses short, broad chelicerae. Multiple weapon morphs in a single species is a previously unknown phenomenon and our findings have significant implications for understanding weapon diversity and maintenance of polymorphism. Specifically, this species will be a valuable model for testing how weapons diverge by being able to test directly for the circumstances under which a certain weapon type is favoured and how weapon shape relates to performance.

Sexual selection has driven the evolution of a spectacular array of male weaponry and alternative reproductive tactics (ARTs) among animals^1^,^2^. Although the profusion of weaponry can be explained by the increased mating opportunities they confer, these benefits do not necessarily explain why there is such an incredible diversity in the form and function of weapons among species. One way to approach this problem is to identify and examine species that exhibit intrasexual variation in weaponry, where weapons can be expressed in more than one exaggerated form; a phenomenon that until now has not been recognised within a single species.

In species that possess weapons, males are commonly divided into two morphs (male dimorphism) – ‘majors’ characterised by heavy investment in weaponry and reliance on aggressive behaviour to secure mates, and ‘minors’ which have reduced weapon size and engage in an ART such as sneaking behaviour. The majority of male dimorphisms are plastic, driven by a conditional strategy^3^, where individual phenotypes are determined largely by environmental conditions experienced during development (e.g. diet quality, climatic stress, parasite load), with pathways such as insulin/IGH and juvenile hormone playing an important role in regulating trait expression^4^,^5^,^6^. Exaggerated traits are honest signals of male condition because these traits are much more sensitive to circulating hormone levels than primary sexual traits (i.e. genitalia) and non-sexual traits (e.g. wings), and hormone titre is directly linked to male condition^7^. Each phenotype is regulated by a threshold, where trait expression results in an abrupt switch in the slope of the scaling relationship between body and weapon size, and this threshold can itself exhibit genetic variation and evolve^8^,^9^. Male trimorphisms in traits used as weapons have recently been identified in beetles and weta, where males can be allocated to one of three morphs by threshold mechanisms at two body size switchpoints^10^,^11^,^12^, but how the morphs correspond to distinct mating tactics is unknown. In these species, weapon size is accurately predicted by body size.

Genetic trimorphisms with distinct male phenotypes have also been identified in species such as side-blotched lizards^13^ and damselflies^14^, where morphs are determined by the inheritance of allele combinations at one or few loci and are not condition-dependent; for any given body size males can exhibit one of a number of forms. Similarly, male Gouldian finches (*Erythrura gouldiae*) exhibit one of three head colours, which influences complex dominance-related interactions among the three morphs^15^, and polymorphism appears to be maintained through frequency-dependent selection. Although these male traits can be used to signal dominance to other males^13^,^14^,^15^,^16^, to our knowledge, no examples of genetic polymorphisms in weaponry (outgrowths used in physical combat) have been described. Furthermore, female choice in combination with male mating strategies are likely to play an important role in maintaining morph frequency in these polymorphic species^17^,^18^. Despite male morphs typically being described by discrete models of either genetic polymorphism or phenotypic plasticity^3^, in reality it is unlikely that the mechanisms are so clear cut, and instead there is likely to be an interplay between genes and the environment^19^.

Here, we describe remarkable weapon polymorphism in a harvestman (*Pantopsalis cheliferoides*: Opiliones: Neopilionidae) with exaggerated chelicerae, where individual males can exhibit one of two exaggerated but uniquely different morphs. Male dimorphism and ARTs have been identified in numerous harvestmen^20^, and are thought to be driven by a conditional strategy^21^,^22^. Our study, however, presents an entirely novel pattern because all previously identified examples of weapon polymorphism in animals describe one armed morph coupled with one or more subordinate morphs that lack weaponry or display greatly reduced forms of the trait.

## Results

### Morphometric analyses

**Figure 1:**
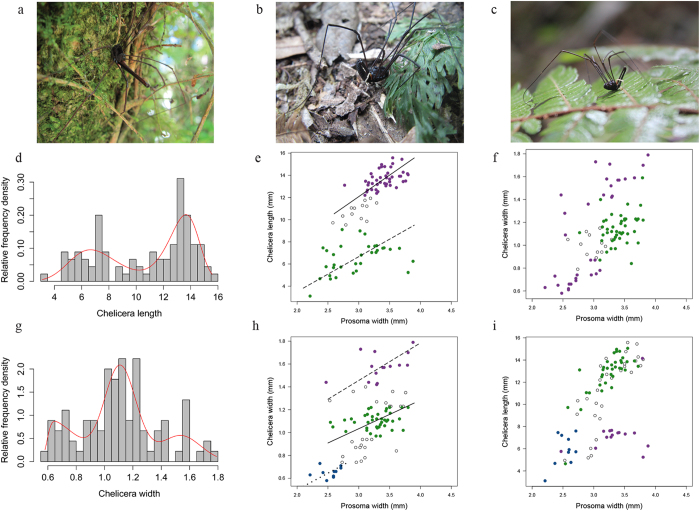
Weapon polymorphism in *Pantopsalis cheliferoides*. Males were assigned to three weapon morphs: (**a**) long-slender, (**b**) short-broad, or (**c**) a short-slender. (**d**) Chelicera length showed dimorphism. (**e**) The scaling relationship between chelicera length and body size revealed two morphs (open circles <95% confidence of morph assignment): a long-slender morph (alpha, green dots, black line) and a short-broad morph (beta, purple dots, dashed line). However, when these data are plotted against chelicera width (**f**), the beta (purple) morph is split into two groups, suggesting that one measurement of chelicera size does not adequately capture the variation in this trait. (**g**) Chelicera width showed trimorphism with three skew-normal distributions, revealing a third, short-slender morph (gamma, blue dots, dotted line) that previously was grouped with the short-broad morph (**h**). (**i**) These three morphs plotted on to a scaling relationship of chelicera length demonstrates a split between long-slender (alpha, green dots), short-broad (beta, purple dots) and short-slender (gamma, blue dots) males.

We found weapon size in male *P. cheliferoides* to be highly variable compared to body size (Table 1). Inspection of the allometry between chelicera length and body size revealed an unusual scaling relationship between these two traits (Fig. 1a–i). Finite mixture models assigned males to one of two morphs based on dimorphism in chelicera length (Fig. 1d–f). The standardised major axis regression slope (SMA) fitted to the two morphs showed that alpha males had a slightly steeper slope than beta males, but the confidence intervals greatly overlap (Table 2). However, when we used chelicera width as a measure of weapon size we found that this trait was trimorphic (Table 3, Fig. 1g–i).The SMA slopes did not vary greatly between the three morphs with wide overlap in the confidence intervals (Table 2). However, the fitted slopes demonstrate males designated to the gamma morph had smaller body sizes than alpha and beta males (Fig. 1h). Most importantly, the slopes show that weapon expression in alpha and beta males is not dependent on body size (Fig. 1h)

### Behavioural observations

**Figure 2:**
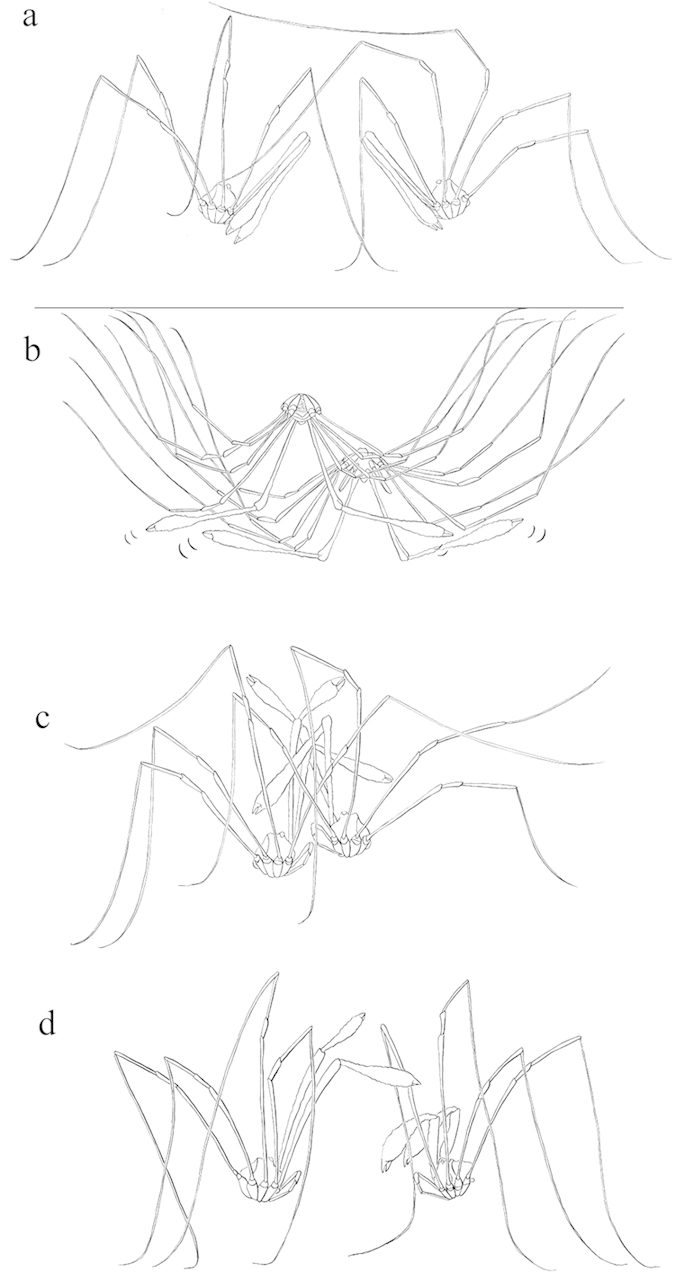
Aggressive interactions between male *Pantopsalis cheliferoides*. (**a**) Interactions between both exaggerated morphs (long-slender and short-broad) are initiated when one male approaches another and touches the opponent with its first and second legs. (**b**) Long-slender males face their opponent, unfold their chelicera so that the second segments are held at right angles to the first, and then rapidly wave their chelicerae in unison, occasionally jabbing towards their opponent. (**c**) In escalated contests males proceed to grappling before one male retreats. (**d**) Rather than completely unfolding their chelicerae, a short-broad male (right) approaches a long-slender male (left), unfolds the second segment to a 45 ° angle and moves his chelicerae up and down in a stabbing movement, also jabbing at his opponent in an attempt to pinch onto their chelicerae.

Behavioural observations of *P. cheliferoides* confirmed that both of the exaggerated morphs use their chelicera as weapons, but in different ways (Fig. 2). While long-slender males extend and rapidly wave their chelicerae in unison with their opponent before engaging in more escalated grappling, short-broad males were observed to punch or stab their chelicerae at their opponent.

## Discussion

We found evidence of two exaggerated chelicera morphs occurring in male *P. cheliferoides*, with behavioural observations to support the hypothesis that both of these unique traits are used as weapons. Furthermore, we identified a third morph (small-slender) of males that had reduced overall chelicera size, suggesting that weaponry in this species is trimorphic. By considering two measures of trait size we were able gain more information and determine that, for any given body size above a threshold, a male can possess one of two forms of exaggerated trait. This is a fascinating pattern because, unlike all other current known examples, weapon expression in this species does not seem to be entirely dependent on body size: male *P. cheliferoides* can possess long-slender or short-broad chelicerae for most body size measures.

We do not yet know how these morphs have evolved and are maintained. Polymorphic male ornaments can be maintained in an evolutionary stable strategy by temporal or spatial variation in directional selection, or via a trade-off between traits that have an intra- or inter-sexual advantage through the use of ARTs^23^. For example, colour polymorphism in *Harmonia axyridis* ladybirds is maintained by temporal variation in female preference for different colour morphs between summer and spring^24^. In systems where polymorphic male ornaments are subject to sexual selection via female mate choice, they may be maintained by frequency dependent selection due to female preference for rare or novel phenotypes. For example, three throat-colour phenotypes in side-blotched lizards (*Uta stansburiana*) fall under an evolutionary stable strategy, where each of three alternative mating strategies beats another strategy but is beaten by a third strategy^13^. Trimorphism is maintained by negative frequency-dependent selection because females prefer to mate with rare morphs to produce high quality offspring which benefit from the rare attributes of their fathers^18^,^25^. We observed ritualised matching of chelicerae by long-slender males suggesting they may assess rivals based on chelicera length, while the jabbing and pinching behaviour of short-broad males may be an effective tactic to disarm rivals. However, we did not observe fighting behaviour by small-slender *P. cheliferoides* males, raising the possibility that they rely on non-aggressive tactics such as sneaking to achieve mating success, and therefore suggesting that the three morphs may use ARTs, with tactics divided between two fighting tactics and one sneaking tactic. We also acknowledge that divergence in chelicera shape could be driven in part by female mate choice, such as for throat colour in side-blotched lizards, in combination with male-male competition.

*Pantopsalis cheliferoides* is not easily assigned to either conditional or allelic trimorphism, presenting a new problem for understanding weapon evolution and diversity. A possible explanation for the maintenance of three morphs is a combination of a condition-dependent facultative threshold and genetic polymorphism. Given that there are two distinct weapon morphs that are not separated by a body size threshold, this strongly suggests that male genotype corresponds to short-broad or long-slender phenotypes. However, the existence of a third (small-slender) morph suggests a second mechanism driving weapon expression, most likely due to a condition-dependent threshold like those seen in most other species with hypervariability in weapon size^21^,^26^. The threshold itself can exhibit genetic variation, resulting in variation between individuals and populations in where the threshold lies along a body size continuum^8^,^9^,^27^. We suggest that a condition-dependent threshold determines weapon size in relation to body size (short-slender versus either long-slender or short-broad), while a genetic polymorphism determines weapon shape for the exaggerated morphs (long-slender versus short-broad). Further investigation of the dynamic interactions of multiple weapon morphs between males may provide important insights into patterns of weapon diversification among species.

## Methods

### Study species

*Pantopsalis cheliferoides* are endemic to New Zealand. They bear chelicerae that are highly sexually dimorphic, being reduced in females but denticulated and extremely exaggerated in males^28^. Male polymorphism occurs in several *Pantopsalis* species, where one morph has long, slender chelicerae, while a second morph possesses club-like chelicerae that are shorter and stouter, with second segments that are more dilated than the first segment (Fig. 1a,b). Chelicera size in both morphs is highly exaggerated and so we determined how this trait relates to body size and its use during competitive interactions between males.

### Measurements

Male *P. cheliferoides* (n = 61) were located and measured in the field at Stubbs Farm (38° 16′ S, 175° 0′ E), west of Waitomo, New Zealand between December 2013 and March 2014. We also measured specimens (n = 29) from the Auckland War Memorial Museum, New Zealand Arthropod Collection and Canterbury Museum. We did not include females because there are currently no descriptions for this genus, and several other harvestmen coexisted at our field site making it difficult to identify the correct species. Using digital callipers (to nearest 0.01 mm), weapon size was measured as the length and width of the second cheliceral segment, while prosoma width was used as an overall measure of body size.

### Statistical analyses

To determine the presence of multiple male morphs we first checked for multimodality in weapon length by inspecting frequency distributions and fitting kernel density estimates. We then fitted finite mixture models and used maximum likelihood ratios to determine morphs^29^,^30^ in the package *mixsmsn*^31^ implemented in R 2.15.3^32^. We ran models that used one, two, or three skew-normal distributions fitted to the weapon size data and then compared them using Akaike Information Criteria (AIC). AIC is calculated as: *2k* – *2ln(l)*, where *k* is the number of model parameters, and *ln(l*) is the natural log of the maximized value of the model’s likelihood function. The model with the lowest AIC score with a difference of at least two AIC scores was considered the most parsimonious. Weaponry was considered dimorphic if it was best described by two skew-normal distributions, or trimorphic if best described by a mixture of three skew-normal distributions. Using the model estimates we assigned individual males to a morph where there was at least 95% probability of correct assignment.

Finally, we calculated the allometric slopes and intercepts (±95% confidence intervals) between chelicera size (length and width) and prosoma width for each male morph using standardised major axis regression in the R package *lmodel2*^33^. We only included individuals which could be assigned to either of the two morphs (for chelicera length) or three morphs (for chelicera width) with more than 95% confidence as calculated using the estimates from the finite mixture models.

### Behavioural observations

We conducted behavioural observations to determine how *P. cheliferoides* use their chelicerae during male-male interactions. Males (n = 28) were collected at Stubbs Farm, bought back to the lab and housed separately in plastic cups half-filled with damp moss and covered with mesh to allow air to circulate. Specimens were fed daily with dry dog food (My Dog Roast Chicken), diced carrots and freshly killed *Tenebrio molitor* larvae, and sprayed twice-daily with water.

Observations took place in 12-inch polyester mesh cube cages (Bioquip) between 2200 and 0500 hours, when *P. cheliferoides* were previously determined to be active in the field (CJ Painting & GI Holwell personal observations). Four replicates of seven male *P. cheliferoides* were randomly chosen and placed in a cage, and we subsequently observed and described how chelicerae were used by males belonging to each morph.

## Additional Information

**How to cite this article**: Painting, C. J. *et al.* Multiple exaggerated weapon morphs: a novel form of male polymorphism in harvestmen. *Sci. Rep.* **5**, 16368; doi: 10.1038/srep16368 (2015).

## Figures and Tables

**Table 1:** Measurements of weapon (chelicera) and body (prosoma) size of male *Pantopsalis cheliferoides.*

	Range (mm)	Mean (standard deviation)	CV%
Chelicera length (mm)	3.11–15.58	10.64 (3.44)	32.37
Chelicera width (mm)	0.58–1.79	1.12 (0.28)	25.49
Prosoma width (mm)	2.21–3.38	3.18 (0.38)	11.97

**Table 2:** Scaling relationships (slopes and intercepts ± 95% confidence intervals) between chelicera size and prosoma width for male *Pantopsalis cheliferoides* using standard major axis regression.

Morph	n	Slope				Intercept	Upper CI
		Estimate	Lower CI	Upper CI	Estimate	Lower CI	
***Chelicera length***	78						
alpha (green)	45	3.87	2.96	5.06	0.46	−3.59	3.55
beta (purple)	33	3.15	2.26	4.39	−2.79	−6.45	−0.17
***Chelicera width***	65						
alpha (green)	40	0.25	0.18	0.34	0.28	−0.02	0.34
beta (purple)	15	0.33	0.20	0.54	0.47	−0.24	0.91
gamma (blue)	10	0.29	0.14	0.61	−0.08	−0.88	0.30

The colours in parentheses refer to those used in Fig. 1.

**Table 3:** Detection of male dimorphism and trimorphism in chelicerae size of *Pantopsalis cheliferoides*.

	Chelicera length		Chelicera width	
	AIC	ΔAIC	AIC	ΔAIC
1 distribution	459.49	20.5	33.06	3.09
2 distributions	**438.99**	**0**	34.84	4.87
3 distributions	443.01	4.02	**29.97**	**0**

Note: The best model is highlighted in bold. ΔAIC is compared to the best model.
